# MRI-Based Computational Model of Heterogeneous Tracer Transport following Local Infusion into a Mouse Hind Limb Tumor

**DOI:** 10.1371/journal.pone.0089594

**Published:** 2014-03-11

**Authors:** Kulam Najmudeen Magdoom, Gregory L. Pishko, Lori Rice, Chris Pampo, Dietmar W. Siemann, Malisa Sarntinoranont

**Affiliations:** 1 Department of Mechanical and Aerospace Engineering, University of Florida, Gainesville, Florida, United States of America; 2 Department of Radiation Oncology, University of Florida, Gainesville, Florida, United States of America; University of Arizona, United States of America

## Abstract

Systemic drug delivery to solid tumors involving macromolecular therapeutic agents is challenging for many reasons. Amongst them is their chaotic microvasculature which often leads to inadequate and uneven uptake of the drug. Localized drug delivery can circumvent such obstacles and convection-enhanced delivery (CED) - controlled infusion of the drug directly into the tissue - has emerged as a promising delivery method for distributing macromolecules over larger tissue volumes. In this study, a three-dimensional MR image-based computational porous media transport model accounting for realistic anatomical geometry and tumor leakiness was developed for predicting the interstitial flow field and distribution of albumin tracer following CED into the hind-limb tumor (KHT sarcoma) in a mouse. Sensitivity of the model to changes in infusion flow rate, catheter placement and tissue hydraulic conductivity were investigated. The model predictions suggest that 1) tracer distribution is asymmetric due to heterogeneous porosity; 2) tracer distribution volume varies linearly with infusion volume within the whole leg, and exponentially within the tumor reaching a maximum steady-state value; 3) infusion at the center of the tumor with high flow rates leads to maximum tracer coverage in the tumor with minimal leakage outside; and 4) increasing the tissue hydraulic conductivity lowers the tumor interstitial fluid pressure and decreases the tracer distribution volume within the whole leg and tumor. The model thus predicts that the interstitial fluid flow and drug transport is sensitive to porosity and changes in extracellular space. This image-based model thus serves as a potential tool for exploring the effects of transport heterogeneity in tumors.

## Introduction

Cancer treatments based on systemic delivery of therapeutic agents are often hindered due to poor and uneven uptake of drugs within tumors. The unique characteristics of the tumor microenvironment which includes irregular microvasculature and high interstitial fluid pressure (IFP) are known to affect the efficacy of anti-cancer treatments such as chemotherapy. The tumor microvasculature characterized by fenestrated, disorganized vessels, necrotic regions and avascular areas [1]–[3] leads to heterogeneous extravasation of therapeutic agents [4], while the high IFP may cause inefficient uptake due to decreased transcapillary transport [5].

In recent years, localized drug delivery has emerged as a plausible alternative to systemic delivery for transporting macromolecular therapeutic agents to the tumors [6]–[11]. By directly injecting into the tumor, this circumvents previously mentioned vascular and interstitial barriers and also reduces side-effects associated with systemic exposure. Amongst the available techniques, convection-enhanced delivery (CED) appears promising because at a given time it can achieve larger distribution volumes than by diffusion alone [12], [13]. In CED, an infusion pump delivers the drug at constant flow rate or pressure thereby creating extracellular fluid flow in tissue, to deliver and distribute macromolecules over larger volumes.

Since its advent, CED has been used for delivery of a wide range of substances including nanoparticles [14], liposomes [6], [15], cytotoxins [16] and viruses [17], [18]. Experimental studies on CED of liposomes into brain tumors (glioma) in rats are encouraging; it was found that the technique effectively distributed liposomes in the tumor and the surrounding normal tissue [6]. On the other hand, a broad heterogeneous distribution was reported to have resulted from CED of cytotoxins into human gliomas [16]. Such an asymmetric distribution was also reported by Boucher and his colleagues in their study with mice involving intratumoral infusion of Evans blue-albumin into sarcoma HSTS 26T [19]. It should however be noted that spherically symmetric distributions for colon adenocarcinoma LS174T were also reported in their study.

Computational modeling of CED has gained attention recently, with pre-clinical and clinical research suggesting the importance of optimization of CED [20], [21]. Software taking into account individual characteristics of a patient's anatomy and pathophysiology for the initial plan of CED is likely to be helpful in deciding catheter placement for optimum distribution volume [20]. However, many current tumor models assume theoretical tumor microvasculature (network based) and simplified tumor geometries [22]–[27]. Eventhough, such models might have the potential to incorporate individual capillary vessels, most of current ones are theoretical and often lack complete transport physics, since accurately reconstructing the entire capillary network and numerically solving for flow physics is computationally intensive. For example, Smith and Humphrey developed a theoretical model for infusions in a spherical tumor with a necrotic core and showed that the flow field was very sensitive to the ratio of vascular conductivity and hydraulic conductivity, and infusion close to the tumor was retarded by the outward flow [22]. Our group has been developing image-based computational porous media models incorporating realistic geometries and spatially-varying transport properties obtained through MRI, for predicting tracer distributions in different tissues [28]–[32]. For tumors in particular, our group developed a framework which accounts for the realistic tumor leakiness by using dynamic contrast enhanced (DCE)-MRI data to estimate the spatial variation of transport properties (rate transfer constant between plasma and extracellular space, 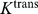 and porosity, 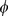) which were included in a porous media model to solve for interstitial fluid flow and tracer transport using computational fluid dynamics techniques [30]–[32].

In this study, a DCE-MRI-based computational model was developed for predicting albumin tracer distribution following CED in the lower limb of a mouse (C3H) inoculated with murine sarcoma cells (KHT), as opposed to systemic delivery which was modeled in our previous studies. Direct injections into the interstitial space have the potential to greatly alter the interstitial fluid pressure and velocity fields, and it is the goal of this study to investigate the role of tissue heterogeneity on CED into tumors. Such a model could potentially help assess the efficacy of CED in tumors and provide better understanding of the biophysical IFP and interstitial fluid velocity (IFV) changes due to CED, which are otherwise difficult to measure experimentally. Also DCE-MRI is likely to improve drug efflux estimates of current software models [33], our model being the first one to use DCE-MRI derived parameters (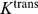 and porosity) to predict CED distributions. Simulations were carried out based on a voxelized modeling approach developed by our group [28], [29], [32]. In this approach, heterogeneous tissue properties (i.e. 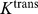 and porosity) and anatomical boundaries are assigned from MRI data. These properties are then incorporated into a porous media transport model to predict CED of tracers. This methodology has been previously used by our group to model CED in spinal cord and brain tissues [28], [29] and systemic delivery in tumors [31], [32]. It should however be noted that the physics, governing equations and resulting physiological flows of the current problem are different. For example, the tumor microenvironment differs significantly from that of the brain due to its aforementioned chaotic vasculature and high IFP. Also porosity dependent formulations for hydraulic conductivity and tracer diffusivity were incorporated in this model, which were not present in our previous studies.

Parameter analysis was performed to study the effects of infusion flow rate, catheter placement and spatially-varying tissue hydraulic conductivity on interstitial fluid flow and albumin tracer transport. This was done to understand the sensitivity of CED distribution to these variables. The flow rate was varied since the capillary fluid exchange is pressure dependent. Catheter placement is also known to be important in CED [13]; studies involving infusions at different locations in the brain have revealed the presence of a optimal site for achieving maximum distribution volumes within a targeted region [29], [34]. The tissue hydraulic conductivity, a measure of fluid conductance through the tissue, was also varied because of its direct influence on tumor IFP and convective flow fields in intratumoral infusions. Higher values of hydraulic conductivity are thought to reduce tumor IFP thereby increasing the filtration of fluids and extravasation of macromolecules [19].

## Materials and Methods

### Mathematical Model

The model requires two components; first, spatially-varying transport properties of the KHT murine sarcoma were estimated using DCE-MRI data following bolus tail vein injection of MR visible tracer gadolinium-diethylene-triamine penta-acetic acid (Gd-DTPA, MW 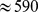 Da) in the mice. The MR experimental data presented in [31] was used in this study. DCE-MRI-derived data was used to determine Gd-DTPA concentration in tissue, rate transfer constant (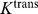) and porosity (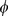) maps as in [31]. Briefly, tracer concentration was calculated based on MR signal enhancement due to the tracer, and the resulting concentration was fitted to the following 2-compartment model (plasma and tissue compartments) to obtain 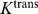 and porosity,
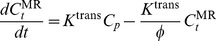
(1)where 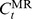 is the MR derived tracer concentration in the tissue, 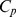 is the tracer concentration in the blood plasma approximated using an arterial input function (AIF) and 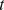 is time. Animal experiments were performed within the principles of the Guide for the Care and Use of Laboratory Animals and approved by the University of Florida Institutional Animal Care and Use Committee (IACUC).

**Figure 1:**
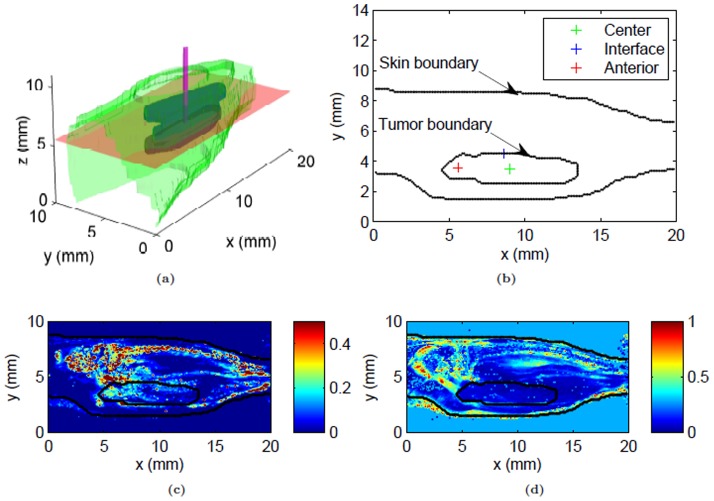
A schematic of the problem along with a sample of the analyzed experimental data. (a) Depiction of baseline CED tumor simulation of tracer spread in hind limb, (b) infusion sites at the mid-plane used in the sensitivity analysis, (c) spatial maps of 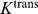 (min^−1^) and (d) porosity at the mid-plane calculated from DCE-MRI data. In (a) the skin boundary is shown in green, tumor boundary in blue, tumor midplane in red and infusion cannula in magenta. The infusion cannula is shown for illustration purpose only.

**Figure 2:**
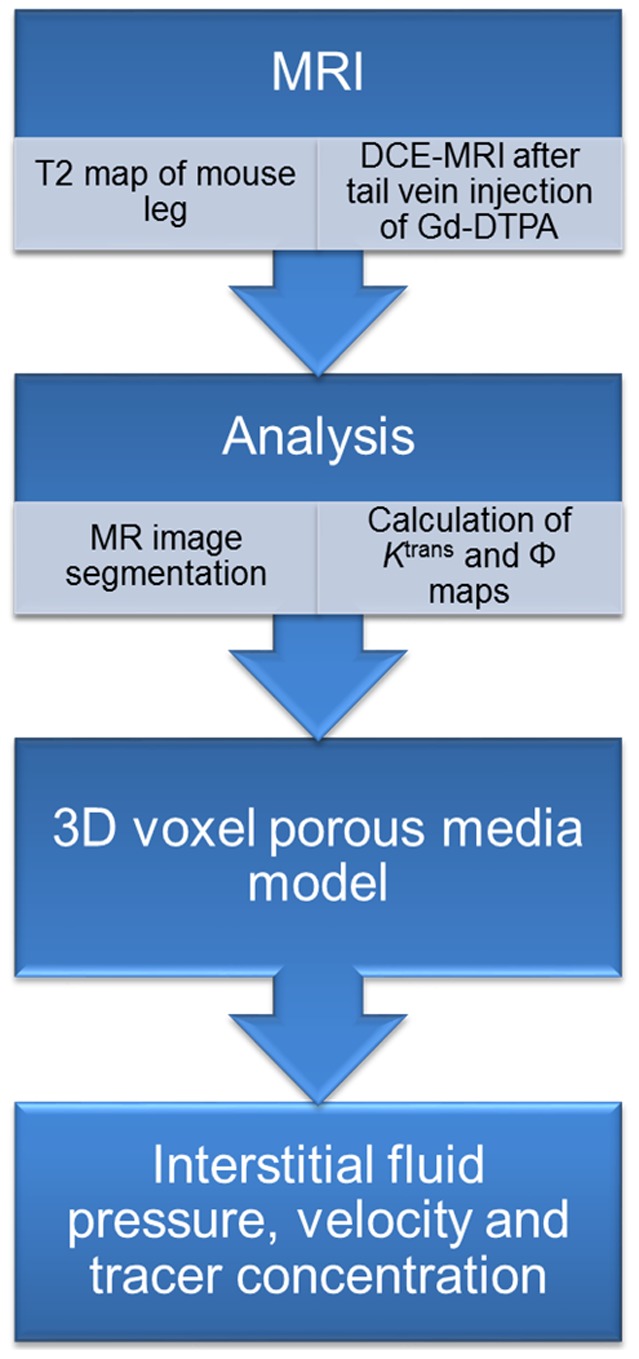
Flow diagram depicting various steps involved in the model.

The second part of the study involves incorporating the above calculated variable transport properties into a computational porous media model for predicting extracellular flow and transport following CED, i.e. direct infusion into the tumor. The CED technique along with a 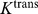 and 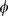 map are shown in Figure 1, and the modeling methods are summarized in the flow chart shown in Figure 2.

The tissue continuum was modeled as a porous media and the governing equations were solved at each voxel after assigning their respective 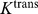 and 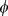 values. For CED, the continuity equation is given by,
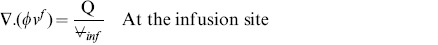
(2a)

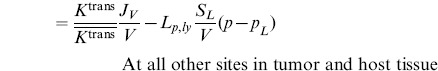
(2b)where 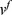 is the fluid phase velocity (convection velocity in the porous medium), 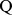 is the CED infusion flow rate of albumin infusate, 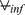 is the volume of the direct infusion voxel, 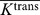 is the average value of 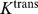 in host and tumor tissue voxels, 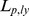 is lymphatic vessel permeability, 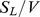 is the lymphatic vessel surface area per unit volume, 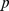 is the IFP and 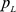 is pressure in the lymphatic vessels which was set to zero. It should be noted that functional lymphatics were assumed only for normal host tissue as tumors lack functional lymphatics (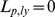) [5]. 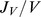 is the filtration rate of plasma per unit volume of tissue into the interstitial space which is given by Starling's law as follows [35],
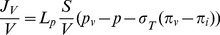
(3)where 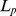 is the permeability of the microvascular wall, 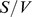 is the blood vessel surface area per unit volume, 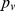 is the vascular fluid pressure, 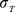 is the osmotic reflection coefficient for plasma proteins, 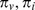 are the osmotic pressures of the plasma and interstitial fluid, respectively.

In the computational fluid dynamics model, properties are assigned on a voxel-by-voxel basis. For voxels that are not infused with albumin infusate, the first term on the right side of the continuity equation (Equation 3) represents the transvascular fluid flux across the microvascular wall per unit volume of the tissue. Assuming similar leakiness patterns for tracer and plasma, this term was scaled by the normalized 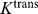 (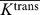) to account for leakiness heterogeneity in the model. The second term accounts for the lymphatic drainage from interstitial space per unit volume of tissue. It should be noted that the IFV in the model is given by 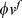.

For a porous medium, the momentum equation is given by Darcy's law,
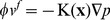
(4)where 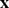 is the position vector, and K is the hydraulic conductivity which is likely to be heterogeneous in tumors and can vary with local changes in porosity of tissues. The following relation, known to predict porosity-dependent variations in hydraulic conductivity for agarose gels and articular cartilage [36], was used
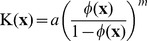
(5a)

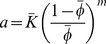
(5b)where 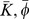 are the average tissue hydraulic conductivity and porosity for tumor or host tissues respectively, and 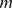 is an empirical exponent which characterizes the sensitivity of hydraulic conductivity to porosity. The scaling factor, 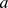 in the relation was calculated based on matching the average tissue hydraulic conductivity with average porosity as shown in Equation 5b.

Albumin tracer transport during CED was modeled using the following convection and diffusion equation with no tissue sources or sinks (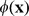 replaced by 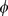 for simplicity), with albumin assumed not to be passively cleared through capillaries within the simulated time points (up to 1 hr).
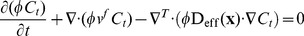
(6)where 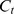 is the concentration of tracer in the tissue. The expression for the effective diffusivity tensor (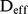) was based on the following empirical relation for diffusion in porous media [37],
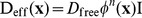
(7)where 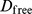 is the self-diffusion coefficient of albumin in water, 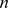 is an empirical exponent set to 1 and 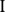 is the Kronecker tensor. The concentration was normalized using the following relation,
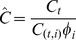
(8)where 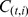 and 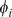 are the infusate concentration and porosity at the infused voxel, respectively. The values of parameters used in the computational model are listed in Table 1.

**Table 1:** Tissue and vascular parameters used in simulations.

Variable	Description	Value	References
*L_p_* (m/Pa.s)	Vessel permeability	2×10^−11*t*^; 3×10^−12*n*^;	[31]
*S*/*V* (m^−1^)	Microvascular surface area per unit volume	20000^t^; 7000^n^	[49]
*L_p,ly_S_L_*/*V* (Pa^−1^s^−1^)	Lymphatic filtration coefficient	1×10^−7^	[31]
*K*_0_ (m^2^/Pa.s)	Baseline hydraulic conductivity	1.9×10^−12*t*^; 3.8×10^−13*n*^	[31]
		7.7×10^−15*e*^	
*p_v_* (Pa)	Microvascular pressure	2300	[31]
*π_i_* (Pa)	Osmotic pressure in interstitial space	3230^t^;1330^n^	[31]
*π_v_* (Pa)	Osmotic pressure in microvasculature	2670	[31]
*σ_T_* (Pa)	Average osmotic reflection coefficient for plasma	0.82^t^; 0.91^n^	[31]
*D*_free_ (m^2^/s)	Self diffusion coefficient of albumin	5.8×10^−11^	[50]

t - tumor,

n - normal tissue,

e - exterior.

MR images also consisted of voxels present outside the mouse which belong neither to tumor or host tissue, i.e. exterior voxels corresponding to surrounding air. In these voxels, the source term for the continuity equation and the diffusivity were set to zero.

### Computational Method

The computational method is identical to the one presented in [32]. Briefly, the continuity, momentum and albumin tracer transport equations were solved using the computational fluid dynamics software package, FLUENT (version 12.0.16, ANSYS, Inc., Canonsburg, PA). For the 3D computational tissue model, a rectangular volume (20×10×9 mm^3^) enclosing the tumor was created and meshed with quadrilateral elements (voxels) of size equal to the MRI resolution (0.104×0.104×1 mm^3^) using the meshing software (GAMBIT, Fluent, Lebanon, NH) with one-to-one mapping between the computational fluid dynamics mesh and MR data. Infusion was carried out at the center of the tumor with a tissue hydraulic conductivity parameter, 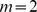 at a flow rate of 0.3 µL/min (baseline case). The value of 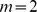 is the baseline value that was used for both tumor and normal tissue. This value was chosen to closely resemble the Carman-Kozeny equation [38] which has been earlier used to describe the hydraulic permeability in tumors [39]. Although the same value of 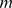 was used for both the tumor and normal tissue, it should be noted that the constant 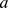 in the relation is different and chosen based on the values reported in literature [31].

### Parameter Analysis

The effects of changing the baseline simulation variables (flow rate, catheter placement and value of 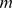) on interstitial flow field and tracer transport were studied separately. The simulation was carried out with two additional flow rates (1 and 3 µL/min) and infusion sites : 1) tumor-host tissue interface and 2) anterior end of the tumor (Figure 1b). The reason for choosing a site at the tumor-host tissue interface was because of the presence of higher convective currents in that region due to the steep decreases in IFP which could result in higher convective velocity. The choice of an infusion site at the anterior end and center of the tumor was to study the distribution at various positions inside the tumor. The effect of changing the tissue hydraulic conductivity was achieved by varying its sensitivity to heterogeneous porosity using the empirical exponent (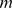). Two additional values of 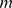 = 5 and 10 were used to simulate the effect of increased tissue hydaulic conductivity. Vessel permeability and diffusivity (varying 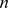) were not included in the sensitivity analysis based on the results of our previous study on transport in tumors [31], where variations in these parameters did not greatly influence tracer transport.

Infusion simulations were carried out up to 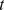 = 1 hr and the interstitial distribution of albumin tracer was quantified at intermittent time points, 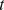 = 15 and 60 mins. Initial conditions for tracer transport assumed no tracer in the tissue, 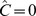, except at the infusion site which is one voxel (0.104×0.104×1 mm^3^). At this site it was set to a normalized value of 1 at all the times during the transport simulation through an user-defined function. The distribution volume was calculated as the volume occupied by voxels having an albumin concentration greater than 15% of the maximum concentration (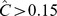). A zero fluid pressure condition, 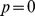, was applied along the cut ends of the tissue volume, and the remaining outer boundaries of the geometry were assigned as a wall boundary, flux = 0. The impermeability condition along the skin boundary was achieved by assigning hydraulic conductivity two orders of magnitude lower than the normal tissue, in the exterior voxels. The assignment of low hydraulic conductivity creates a material that is less penetrable and resistant to fluid motion. For the chosen value of hydraulic conductivity at the exterior voxels, the maximum mean velocity at the skin boundary for all the simulations was calculated to be close to zero (0.02 µm/s).

## Results

### Baseline case

**Figure 3:**
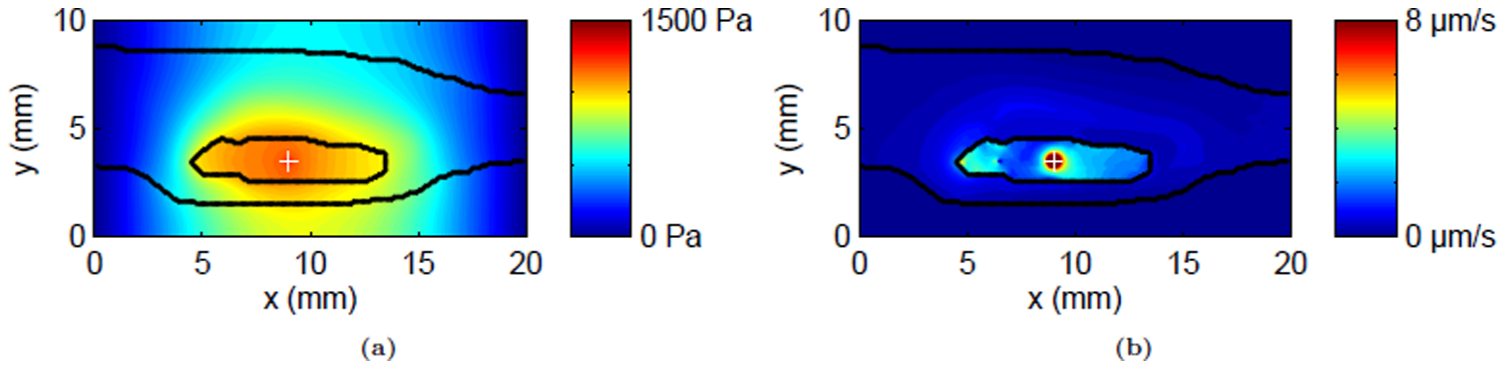
Predicted flow field with local infusion (0.3 µL/min) at the center of the tumor. The interstitial fluid pressure (IFP) and convective fluid velocity within the plane of infusion is shown by its contours (a & b respecively). Tumor and skin boundaries are overlaid on the contours; the infusion site is shown by a plus sign.

The predicted IFP inside the tumor was elevated independent of CED as shown in [32]. With CED at 0.3 µL/min, the pressure at the infusion site was increased by approximately 0.33 kPa (≈21% increase) in addition to the overall elevation inside the tumor as shown in the contour plot (Figure 3a). CED created additional pressure gradients (≈0.65 kPa/mm along the infusion plane) around the infusion site which were absent with systemic delivery. There was also significant pressure gradients at the anterior edge of the tumor-host tissue interface (≈0.67 kPa/mm) which was also observed without CED [32]. It should be noted that the pressure gradient outside the skin does not contribute to the extracellular fluid flow due to the very low hydraulic conductivity assigned in those voxels.

The convective velocity is shown in Figure 3b. The predicted velocity field was heterogeneous with maximum velocity near the infusion site (35 µm/s). There were also significant outwardly-directed flows at the tumor-host tissue interface especially near the anterior end of the tumor (≈0.45–3.2 µm/s) which was also observed with systemic delivery [32]. Thus CED has resulted in alterations of endogenous flows closer to the infusion site to a far larger magnitude.

**Figure 4:**
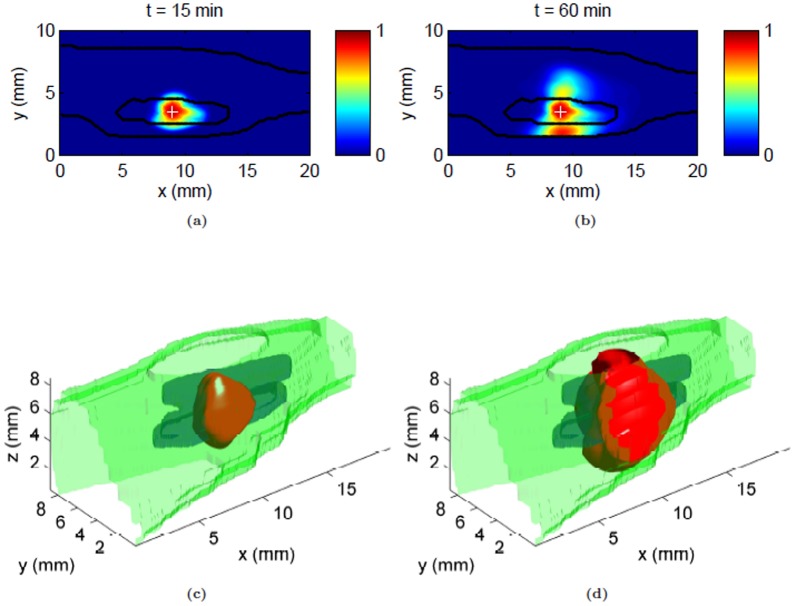
Concentration profile following CED of albumin infusate (0.3 µL/min) at the center of the tumor. (a & b) Normalized albumin tracer concentration contours within the infusion plane at 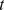 = 15 and 60 min respectively. Tumor and skin boundaries are overlaid on the contours; the infusion site is designated by a plus sign. (c & d) Predicted 3D distributed volume at 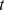 = 15 and 60 min respectively, shown by an iso-surface of the distribution volume (threshold, 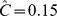). Visible boundaries are for tumor (blue), skin (green) and distribution volume (red).

**Figure 5:**
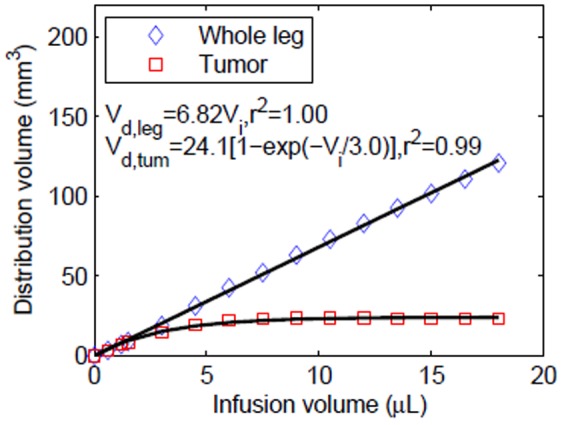
Tracer distribution volumes in tissue for varying infusion volume within the whole leg (tumor and surrounding tissue) and tumor only, following CED at 0.3 µL/min. Distribution volume dependence on infusion volume was fit to linear (whole leg) and exponential (tumor only) models.

Contours of normalized albumin concentration at various time points following CED infusion are shown in Figures 4a & 4b. Albumin distribution around the infusion site was asymmetric for all the times simulated with high concentration inside the tumor. This is in contrast to systemic delivery [31], [32] where the tracer distribution was heterogeneous with high concentration regions outside the tumor. The effect of the skin boundary near the tumor on the distribution pattern was evident at later time points, with gradually increasing tracer accumulation along the skin boundary. Iso-surfaces of the distribution volume at intermittent time points, shown in Figures 4c & 4d, depict the evolution of the concentration profile with time. They show the non-uniform nature of the distribution and the tangential flux of albumin tracer along the skin boundary near the tumor. For the chosen threshold value, albumin was distributed to approximately 48% of the tumor volume after one hour of infusion at 0.3 µL/min. The variation of distribution volume (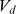) with infusion volume (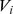) within the whole leg and tumor, in particular is shown in Figure 5. As expected, plots show a linear relationship between distribution volume and infusion volume for the whole leg; however, a more exponential variation was observed within the tumor and the resultant distribution volume reached a maximum steady-state value in this targeted tumor region (≈48%).

### Parameter Analysis

**Figure 6:**
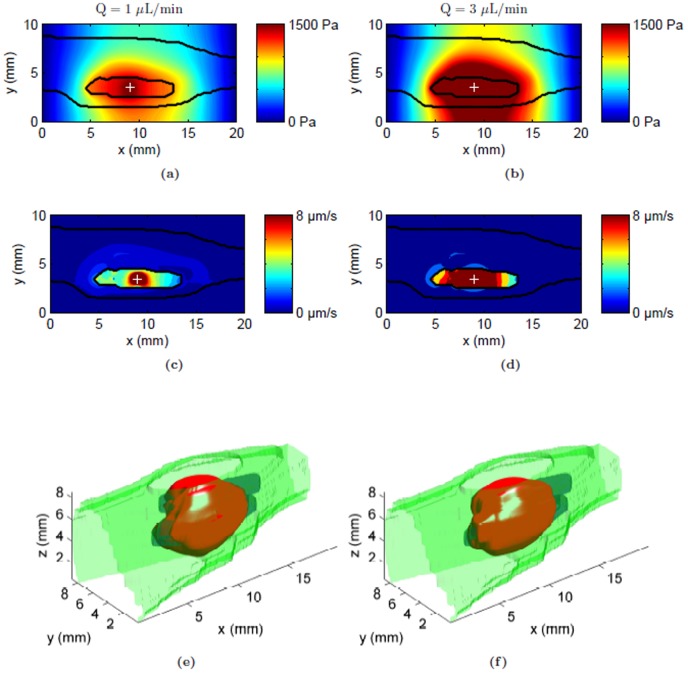
Flow field and tracer distribution following CED of albumin tracer at the center of the tumor for two different flow rates: 1 µL/min and 3 µL/min. (a & b) Interstitial fluid pressure (IFP) contours for 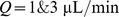 respectively, and (c & d) convective fluid velocity contours at the infusion plane for 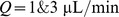 respectively. Tumor and skin boundaries are overlaid on the contours; the infusion site is designated by a plus sign. (e & f) Predicted 3D tracer distribution volume at the end of infusion (1 hr) shown by an iso-surface of the distribution volume threshold (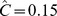) for 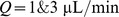 respectively. Visible boundaries are for tumor (blue), skin (green) and distribution volume (red).

**Figure 7:**
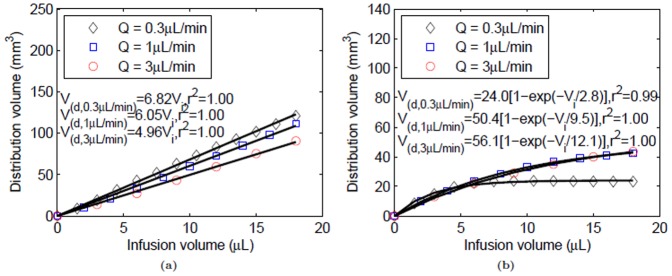
Variation of tracer distribution volumes in tissue with infusion volume for the whole leg and tumor following CED at center of the tumor for three different flow rates (0.3, 1 and 3 µL/min). Distribution volume dependence on infusion volume was fit to (a) linear (whole leg) and (b) exponential (tumor only) models.

With higher infusion flow rates of 1 and 3 µL/min, IFP close to the infusion site increased by approximately 58% and 226% from the baseline value, respectively (Figures 6a & 6b). With increasing flow rates, convective velocity became orders of magnitude higher inside the tumor than outside (Figures 6c & 6d). The peak fluid velocity values predicted close to the infusion site increased by approximately 228% and 900% from the baseline for infusion flow rates of 1 and 3 µL/min, respectively. These changes were reflected in the predicted tracer distribution in the tissue shown as isosurfaces in Figures 6e & 6f. Increasing the flow rate tended to confine the tracer more within the tumor and reduced spread into adjacent normal tissue. It also reduced the tangential flux of the tracer along the skin boundary close to the tumor. As expected, the distribution volume within the whole leg increased linearly with the infusion volume. However for a given infusion volume, the distribution volume within the whole leg decreased with increasing flow rates (Figure 7a). Within the tumor, higher flow rates resulted in more coverage (Figure 7b) with 85% of the tumor being covered by the tracer infused at a rate of 1 µL/min, and 87% with 3 µL/min for the same amount of infusion volume.

**Figure 8:**
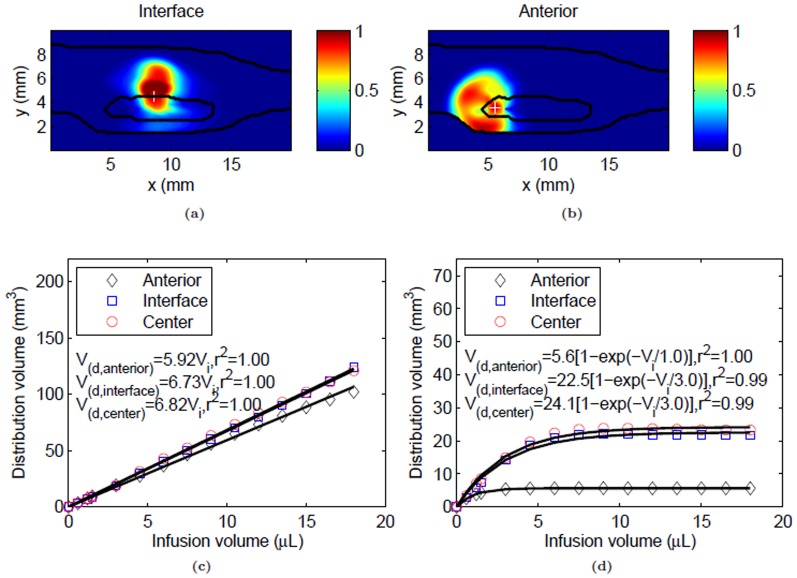
Tracer distribution following CED of albumin (0.3 µL/min) at the tumor-host tissue interface and anterior end of tumor. (a & b) Normalized tracer concentration contours in the infusion plane at the end of infusion (1 hr) in the tumor-host tissue interface and anterior end of tumor respectively. Tumor and skin boundaries are overlaid on the contours, and the infusion site is shown by a plus sign. Variation of tracer distribution volumes in tissue with infusion volume for the (c) whole leg and (d) tumor. Distribution volume dependence on infusion volume was fit to linear (whole leg) and exponential (tumor only) models.

The effect of catheter placement on albumin distribution is shown in Figures 8a & 8b. Asymmetric distributions were observed for infusions at different locations: tumor-host tissue interface and anterior end of the tumor. Infusion at the interface and anterior end of the leg tended to distribute albumin dorsally and anteriorly within the leg, respectively. For the whole leg, tracer spread again exhibited a linear relation between 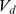 and 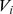, with highest distribution volume for infusion at the tumor center (Figure 8c). Within the tumor, the 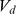 variation with 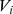 was also approximately exponential with infusion at the tumor center covering the maximum volume (≈48%) while infusion at the anterior end covered less of the tumor, approximately 11% (Figure 8d).

**Figure 9:**
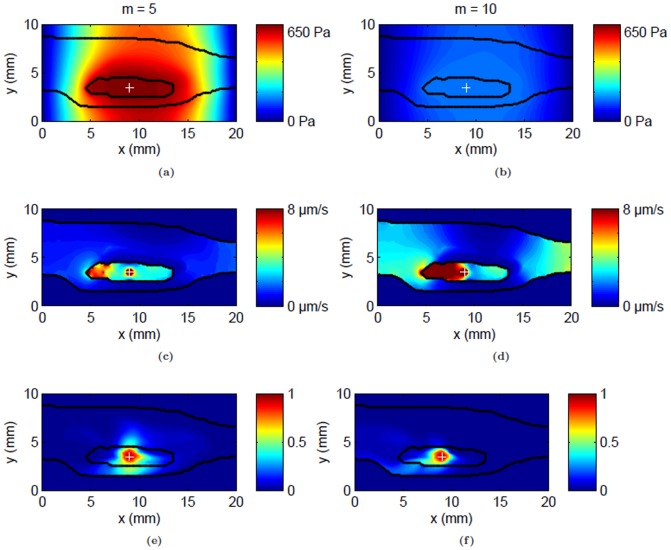
Flow field and tracer distribution following CED of albumin (0.3 µL/min) at the center of the tumor for varying values of the hydraulic conductivity parameter, 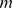. (a & b) Interstitial fluid pressure (IFP) contours for 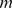 = 5 & 10 respectively; (c & d) Convective fluid velocity contours for 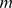 = 5 & 10 respectively; (e & f) Normalized tracer concentration contours at the end of infusion (1 hr) for 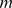 = 5 & 10 respectively. All contours are for the plane of infusion with the infusion site designated by a plus sign.

For varying hydraulic conductivity sensitivity (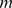 = 5 and 10), the model predicted elevated IFP patterns inside the tumor similar to the baseline results albeit with different peak pressure values (Figures 9a & 9b). The simulation results indicated a 50 and 89% reduction in the peak IFP from the baseline value for 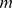 = 5 and 10, respectively. The convection velocity became more heterogeneous with increasing 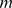 (Figures 9c & 9d). The increase in 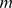 advected fluid away from the tumor particularly through the anterior end.

**Figure 10:**
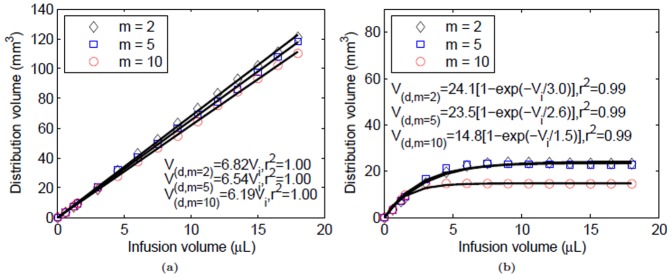
Variation of tracer distribution volumes in tissue with infusion volume for the whole leg and tumor following CED (0.3 µL/min) at the center of the tumor for varying values of the hydraulic conductivity parameter, 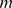. Distribution volume dependence on infusion volume was fit to (a) linear (whole leg) and (b) exponential (tumor only) models.

Changes in the tracer distribution over time at different values of 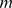 are shown in Figures 9e & 9f. Convective effects influenced the distribution pattern with tracer being transported away from the tumor, particularly towards the anterior cut end of the hind limb. This was more apparent for higher values of 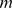. The distribution volume varied linearly with the infusion volume for the whole leg, and exponentially within the tumor for both the values of 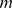 (Figure 10). With larger values of 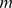, there was also a reduction in the final distribution volume from baseline values (approximately by 2.5 and 2.15% with 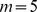, and 9 and 37% with 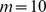 for the whole leg and tumor, respectively) due to exit of tracer at cut ends. Within the tumor, the distribution volume tended to reach a steady-state value more rapidly with increasing values of 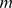 (Figure 10b). One hour of infusion at 0.3 µL/min and 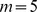 resulted in coverage of approximately 47% of the tumor volume. Whereas for 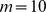, approximately 30% of the tumor volume was covered.

## Discussion

A MR image-based computational model (voxelized model) for predicting distribution of a macro-molecular protein tracer following CED in a mouse tumor was developed. The key advancement and contribution is that our model incorporates vasculature as a realistic and heterogeneous entity, which is novel to CED models. By non-invasively probing the vasculature of the tumor and surrounding area, and incorporating heterogeneous transport into our model we have made some interesting discoveries. 1) Penetration of tracer/drug into surrounding tissue was found to be highly dependent on flow rate, tissue boundaries, vascular leakiness and tissue lymphatic clearance which slowed interstitial flows. The predicted tracer distribution was asymmetric, and increasingly confined within the tumor with increasing infusion flow rate. Such distributions can not be captured with spherical tumor models; 2) Our results also make clear and stress the interconnectivity between hydraulic conductivity, vascular leakiness, and tumor interstitial fluid pressure, the delicate balancing act of treatments that target these mechanisms, and the consequences of doing so in relation to CED. The model predicts lower tumor interstitial fluid pressure and tracer distribution volume for increasing the tissue hydraulic conductivity. Thus, if CED is to be used in conjunction with a therapy aimed at lowering IFP, then it may be best to do so after the CED procedure to ensure a large distribution volume of CED-administered drug. Since these results may vary with varying patient-specific pressure patterns; the model results suggest the importance of conducting CED with a priori knowledge of the interstitial pressure patterns. They can be potentially derived from MRI-based computational modeling methods such as those created by our group [31].

Without CED, the predicted IFP reflected previous experimental findings which have shown elevated relatively uniform pressures inside the tumor [40]–[44]. With CED, the infusion induced an additional local pressure gradient thereby conveying the advantage convection gives in distributing molecules to larger tissue volumes with infusion. Except for near the infusion site, the pressure was relatively uniform inside the tumor and dropped steeply at the periphery which is in agreement with previous experimental findings [5]. Outside the tumor, the tissue boundary condition played a critical role in determining IFP. The close proximity of the tumor to the impermeable skin boundary increased the IFP near its surface approximately by a factor of two (for the baseline case), than at the skin boundary farther from the tumor.

The model predicted heterogeneous convective velocity due to spatially varying pressure gradients induced by CED, porosity and 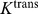 induced by CED. The resulting flow directions reflected the IFP gradient field. High velocities at the infusion site and the anterior end were due to the pressure gradients created due to infusion, and higher leakiness in the region exhibited as increased 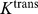 (Figure 1c). Overall, CED altered the extracellular fluid flows inside the tumor especially in the vicinity of the infusion site.

The model was also able to predict asymmetric distribution of tracer conforming with the previous experimental findings [16], [19], [45]. The distribution pattern was closely interlinked with the predicted flow field with high concentration at the infusion site, and gradual spread into the adjacent normal tissue. Such a focal CED distribution of the tracer is in contrast with the one obtained systemically [31], thus making CED delivery a possible alternative to systemic routes. The tracer spread along the skin boundary is due to the close proximity of tumor with the skin. The distribution volume plots (Figure 5) show that the tumor was not entirely covered by the tracer even with longer infusion times. Such a low tumor coverage by the tracer was due to the relatively uniform IFP within the tumor except in the region close to the infusion site (Figure 3a) which restricted the resulting convective velocity (Figure 3b) and tracer filtration within the tumor and directed transport outwards.

Increases in infusion flow rate had profound effects on the tracer distribution as convective velocity outside the tumor was greatly reduced compared to flows enhanced inside the tumor. This was because increasing the flow rate mainly affected the fluid flows locally close to the infusion site. Although the convective velocity is uniform inside the tumor, it dropped steeply at the tumor periphery (Figure 6d) due to greater lymphatic uptake of interstitial fluid in the normal tissue. Thus the tracer could be expected to be convectively transported well inside the tumor but mainly diffusely at the boundary. This combined with the lack of lymphatics inside the tumor caused more tracer to distribute within the tumor than outside, and this effect became more pronounced at higher flow rates. For the same amount of infusion volume, the tumor was almost fully covered by the tracer at a flow rate of 3 µL/min. The challenge regarding using high infusion rates experimentally is backflow, which our model did not account for. Specially designed cannulas may allow for such infusions at higher flow rates (up to 50 µL/min) without backflow [46], [47].

The sensitivity analysis was also used to study tracer distribution at different catheter positions, in an attempt to find a suitable placement which could maximize distribution volume in the target site. For the set of baseline parameters, we found that infusion at the center of the tumor produced the maximum distribution volume within the tumor. Infusion at the tumor-host tissue interface tended to distribute the drug outside the tumor due to enhanced convective effects at the tumor boundary. It should be noted that, the outward flow of albumin from the tumor for infusions at the anterior end of the tumor is also partially due to an unphysical artifact, which is the proximity of the infusion site to the cut ends of the tumor (due to limited field of view) where a zero pressure boundary condition was specified. A similar pattern can be expected for infusions at the posterior end of the tumor. In the future, this approach can be automated to solve infusion in every voxel to determine the optimal infusion site thereby helping with surgical planning on a case-by-case basis.

The possibility of reducing the tumor IFP by increasing the sensitivity of tissue hydraulic conductivity to tissue porosity was also investigated. This analysis was done to partially account for soft tissue swelling and resulted in increased heterogeneous transport. Also several studies have explored the idea of reducing the tumor IFP as a way to overcome the drug delivery barriers [5]. For example, compounds such as VEGF inhibitors, hyaluronidase, mannitol among others have been used to disrupt the heterogeneous tumor microvasculature in an attempt to lower tumor IFP and improve drug delivery [5], [48]. By doing so, the underlying tissue hydraulic conductivity may also change [19] and it might be important to study how this affects the drug distribution. Mathematically, this was implemented by varying the empirical parameter 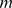 in the expression for tissue hydraulic conductivity (Equation 5a). The parameter 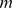 could be thought of as a variable to either amplify or reduce the fluid flows in the heterogeneous pathways in the whole leg determined by the 2-compartmental model. Increasing 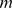 caused more interstitial fluid to leak away from the tumor periphery and reduced the tumor IFP as shown in Figure 9. Increasing the hydraulic conductivity has been previously shown to reduce IFP and thus increase extravasation of macromolecules [19]. The results of this sensitivity analysis indicated reduction in peak tumor IFP compared to baseline simulation, however the resulting CED tracer distribution volumes were also reduced. This was because of increase in convective velocity heterogeneity resulting from very high reduction in the tumor IFP, which directed the interstitial fluid and albumin tracer away from the tumor into adjacent normal tissue. The amplified hydraulic conductivity in the whole tissue volume opened up various low resistant fluid pathways through which the tracer got transported away from the tumor. High interstitial velocity at the anterior cut end compared to the posterior side is due to the high 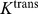 described in the earlier paragraph. These results demonstrate the importance of transport heterogeneity and measuring extracellular transport, especially changes in extracellular space for a given tumor, in order to achieve improved understanding of spatial drug distribution within the tumor.

In this study, an image-based tumor model was developed which incorporates realisitic tumor vascular leakiness with anatomical geometries, and used to predict heterogeneous/asymmetric drug distribution following direct infusions. Although the results discussed in this study are restricted to the mouse hind limb tumor under study, it should be noted that the applicability of such a voxelized model to a wide range of tumors is possible. With further experimental validation and measure of tissue properties, this model could be potentially applied towards patient-specific treatments and to more accurately investigate effects of flow patterns on heterogeneous tumor drug delivery. The model however has some limitations at present: 1) model parameter values such as average hydraulic conductivity, diffusivity, scaling factor for the leakiness term (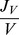) obtained from literature varies across tumors and needs to be determined experimentally for a given tumor; 2) modeling heterogeneous tumor microvasculature based on fluid exchange between blood plasma and tissue compartments is only approximate since one assumption is that each MR voxel consists of tissue and blood vessels. Hence at a sufficiently high MR resolution, model predictions could be erroneous in bigger blood vessels, highly vascularized and necrotic regions; and 3) tracer backflow along the infusion needle has not been accounted for. However, even with these limitations, the model is still able to capture major characteristics of heterogeneity and provide important insights into CED tracer transport in tumors.
